# Sirtuin 1 serum concentration in healthy children - dependence on sex, age, stage of puberty, body weight and diet

**DOI:** 10.3389/fendo.2024.1356612

**Published:** 2024-03-11

**Authors:** Anna Fedorczak, Andrzej Lewiński, Renata Stawerska

**Affiliations:** ^1^ Department of Endocrinology and Metabolic Diseases, Polish Mother’s Memorial Hospital – Research Institute, Lodz, Poland; ^2^ Department of Paediatric Endocrinology, Medical University of Lodz, Lodz, Poland

**Keywords:** sirtuin 1, growth, puberty, IGF-1, diet, healthy children

## Abstract

**Introduction:**

Sirtuin 1 (SIRT1) is known to be involved in sensing cellular energy levels and regulating energy metabolism. This study aimed to evaluate fasting serum SIRT1 levels in healthy children, and to analyse the influence of age, sex, puberty, body weight, height, and diet on its concentration.

**Methods:**

47 healthy children aged 4-14 with weight and height within normal range and no chronic disease were included into the study. Fasting serum SIRT1 concentrations were estimated by Enzyme Linked Immunosorbent Assay (ELISA).

**Results:**

Results showed that serum SIRT1 concentrations in healthy children did not differ with respect to sex, age, height, weight and puberty. Whereas, it appeared that a higher frequency of fruits, vegetables and dairy products consumption was associated with an increase in serum SIRT1 levels.

**Discussion:**

Studying SIRT1 in the context of children’s health may have implications for a broader understanding of growth processes, pubertal development, metabolic disorders and nutrition.

## 1 Introduction

The sirtuins are a family of nicotinamide adenine dinucleotide (NAD+)-dependent deacylases that regulate many cellular processes (1). Among the seven currently known types of sirtuins, sirtuin 1 (SIRT1) is involved in the cellular signal transduction and metabolism, DNA repair, inflammation as well as regulation of cellular senescence and aging (2–11). Despite numerous experimental studies on the importance of SIRT1 in the human body, there is very limited data on the level of blood concentration of this protein in humans. It should be noted that SIRT1 is directly engaged in intracellular GH signal transmission for IGF-1 secretion via modulation of JAK2/STAT pathway, also in the regulation of GH release in the central nervous system, as well as growth-plate chondrogenesis and longitudinal bone growth (12–15). Thus, it can be assumed that the concentration of SIRT1 should vary in dependence on age and growth rate in children.

On the other hand, it is known that changes in SIRT1 concentration affects the function of the hunger and satiety center, and the use of sirtuin activators promotes weight loss in obese individuals (16). Whereas in a fasting state, caloric restriction or malnutrition, SIRT1 intensifies catabolic processes and inhibits anabolic processes to maintain homeostasis (17–19). SIRT1 is also involved in regulating puberty (20). Moreover, there are some natural substances contained in daily consumed foods that act as sirtuin triggers (21–24). Thus, its concentration also probably varies depending on the body mass, stage of puberty, as well as diet.

The aim of this study was to evaluate fasting serum SIRT1 concentration in healthy children, and to analyse the influence of age, sex, body height, body mass and stage of puberty, as well as dietary habits and type and amount of nutrients intake.

## 2 Materials and methods

### 2.1 Study group

From among the children admitted to the Polish Mother Memorial Hospital - Research Institute in Lodz, Poland, the study group included those children who did not have any known healthy problems and who did meet the inclusion criteria and did not meet the exclusion criteria.

Inclusion criteria:

aged: 4-16 years, height and weight in the reference range (3rd-97th percentile for age and sex based on local percentile charts), completing a nutritional questionnaire, written consent of the legal representative to participate in the study.

Exclusion criteria:

chronic health problems which may influence the results (e.g. chronic diseases of the gastrointestinal tract, respiratory system, circulatory system, endocrine system, anorexia nervosa, undernutrition, obesity, short stature, excessive height, genetic disorders and syndromes), acute illness, no written consent of the legal representative to participate in the study.

Finally, 47 children were enrolled into the study group.

### 2.2 Auxological assessment

In each child: a detailed medical history was collected and a physical examination was performed. Height and weight measurements were taken in the morning on the day of hospital admission by the clinicians involved in this study (AF or RS). Children’s height was measured with an accuracy of 1 mm using a Harpenden stadiometer. Children were measured without shoes, with their heads in the Frankfort plane and their feet together. The measurement was performed three times and the average value was taken. Body weight was measured using an electronic scale, with an accuracy of 100 grams. During the measurement, the child was in underwear. Based on results of body height and weight, the standard deviation scores (SDS) in relation to the reference values for age and sex were calculated: for height - height standard deviation score (height SDS) and for body mass - weight SDS. Also, the body mass index (BMI) was calculated and expressed as BMI SDS with respect to the reference values for age and sex for Polish population (25, 26). The puberty stage was assessed according to Tanner scale (27).

### 2.3 Laboratory methods

The blood samples were taken in fasting state in the morning, to measure the routinely determined basic biochemical parameters as well as the concentrations of IGF-1 and IGFBP-3. An additional blood sample (2.4 ml) was taken in fasting state in the morning, for the determination of SIRT1 serum concentration. After collection, the blood was centrifuged to obtain serum. The serum with no signs of haemolysis was then frozen and stored at the appropriate temperature (see below), as required by the test manufacturer, until SIRT1 analysis.

All measurements were performed at the Centre for Medical Laboratory Diagnostics and Screening of the Polish Mother’s Memorial Hospital – Research Institute in Lodz, Poland.

SIRT1 concentration was determined by double-binding immunoenzymatic assay (ELISA) using 2 Human NAD-dependent deacetylase Sirtuin-1 (SIRT1/SIR2L1) ELISA Kits (Cusabio, Houston, TX, USA), according to the manufacturer’s instructions (User Manual; catalogue number: CSB-E15058h). The concentration of each sample was measured in duplicate. The sensitivity of the assay is 0.03 ng/ml, while the manufacturer’s guaranteed detection range of the assay is 0.15 ng/ml - 10 ng/ml, with an intra-assay coefficient of variation of less than 8% and an inter-assay coefficient of variation of less than 10%.

IGF-1 and IGFBP-3 concentrations were assessed using Immulite, DPC assays. For IGF-1, the WHO NIBSC 1st IRR 87/518 standard was used, with an analytical sensitivity of 20 ng/mL, a calibration ranges up to 1600 ng/mL, an intra-assay coefficient of variation: 3.1-4.3% and inter-assay coefficient of variation CV: 5,8-8,4%. The assay to assess IGFBP-3 was calibrated to the WHO NIBSC Reagent 93/560 standard, with an analytical sensitivity of 0.02 μg/mL, a calibration ranges up to 426 μg/mL, an intra-assay coefficient of variation of 3.5-5.6%, and an inter-assay coefficient of variation of 7.5-9.9%. IGF-1 concentration was expressed in terms of standard deviation for sex and age (IGF-I SDS), according to reference data (28). The molar ratio of IGF-1/IGFBP-3 was calculated assuming a molecular weight for IGF-1 of 7.5kDa and for IGFBP-3 of 42.0 kDa. The molar ratio of IGF-1 to IGFBP-3 is considered an indicator of the bioavailability of IGF-1 (29).

### 2.4 Assessment of children’s dietary habits and type and frequency of nutrients intake

The assessment of the children’s dietary habits was carried out through dietary survey with parent, wherein the frequency of their offspring’s consumption of particular food categories over the preceding month was examined. The survey was administered by a physician-researcher. The survey was developed based on the CoCu Questionnaire validated on a population of german children (30). After obtaining permission from the authors to use the questionnaire, it was translated bilaterally, and then both versions were checked for compatibility. The survey consisted of two parts. The first part contained 14 questions about the composition of the diet and the second part of the questionnaire contained questions about eating habits and food culture. The parent was asked to rate how many servings of various foods the child consumes per day (fruits or vegetables, unsweetened dairy products, sweetened dairy products, sweetened beverages, whole wheat bread, white bread) or per week (meat, fish, french fries, potatoes, rice or pasta, ready meals, pastries, sweet or salty snacks). Reference portions were described in the text or illustrated with photos. The selection of food items was largely based on the Food Frequency Questionnaire FFQ and the healthy eating pyramid (31, 32). The second part of the survey included questions about eating habits (i.e. whether they follow any specific diet, number of meals they have per day). The questionnaire was included in the **Supplementary Materials**.

### 2.5 Statistical analysis

Statistical analysis of the collected data was performed using STATISTICA ver. 13.3 software (Statsoft, Poland). The Shapiro-Wilk test was used to assess normality of distribution, and the Levene’s test was used to assess equality of variance. Comparative analysis was performed using non-parametric tests for independent variables. Non-parametric The Kruskal–Wallis test by ranks and Mann-Whitney U test were used for intergroup comparisons of quantitative continuous variables. Intergroup comparisons of nominal/qualitative variables were performed using the Chi-square test. In addition, a correlation analysis of the variables was performed (Pearson’s correlation coefficient). Continuous variables were presented median and interquartile ranges (median (Q1-Q3)) and range, categorical variables by N (%)). Statistically significant differences were taken as p-values below 0.05.

### 2.6 Ethics approval

Approval was obtained from the Bioethics Committee at the Polish Mother’s Memorial Hospital – Research Institute in Lodz (Opinion No. 47/2020).

### 2.7 Informed consent statement

The legal representatives of all patients gave their informed written consent to participate in the study prior to their inclusion in the study.

## 3 Results

### 3.1 Study group characteristics

There were 47 healthy children included in the study. Mean age of children 10.35 ± 2.6 years, 57,5% were male. Study group characteristics is presented in **Table 1**.

**Table 1:** Study group characteristics.

Variable	Mean ± SD Median (Q1 – Q3)	Range
age [years] M F	10. 4 ± 2.6 10.6 (8.37 – 12.78) 10.8 ± 2.7 9.7 ± 2.4	4.2 – 14.4 4.2 – 14.4 5.3 – 13.9
sex M; N (%) F; N (%)	27 (57.5%) 20 (42.5%)	
Tanner stage 1; N (M, F) ≥2; N (M, F)	24 (15, 9) 23 (12, 11)	
height SDS	0.52 ± 1.02 0.48 (-0.4 – 1.1)	-1.07 – 2.9
weight SDS	0.48 ± 1.3 0.26 (-0.4 – 1.4)	-2.08 – 3.5
BMI SDS	0.18 ± 1.3 -0.05 (-0.8 –1.1)	-2.25 – 2.8
IGF-1 [ng/ml]	270.5 ± 183.4 203.9 (119.5 – 404.2)	36.6 – 679.4
IGF-1 SDS	-0.39 ± 1.14 -0.16 (-1.33 – 0.47)	-3.66 – 1.41
IGFBP-3 [ng/ml]	4316.6 ± 1434.9 4721 (2905 – 5438)	1542 – 6336
IGF1/IGFBP-3 m.r.	0.32 ± 0.16 0.24 (0.19 – 0.43)	0.11 – 0.7

M, male; F, female; SDS, standard deviation score; BMI, body mass index; IGF-1, insulin like growth factor 1; IGFBP-3, IGF-binding protein 3; m.r., molar ratio.

### 3.2 The analysis of serum SIRT1 concentration in healthy children in dependence on age, sex and stage of puberty

**Figure 1:**
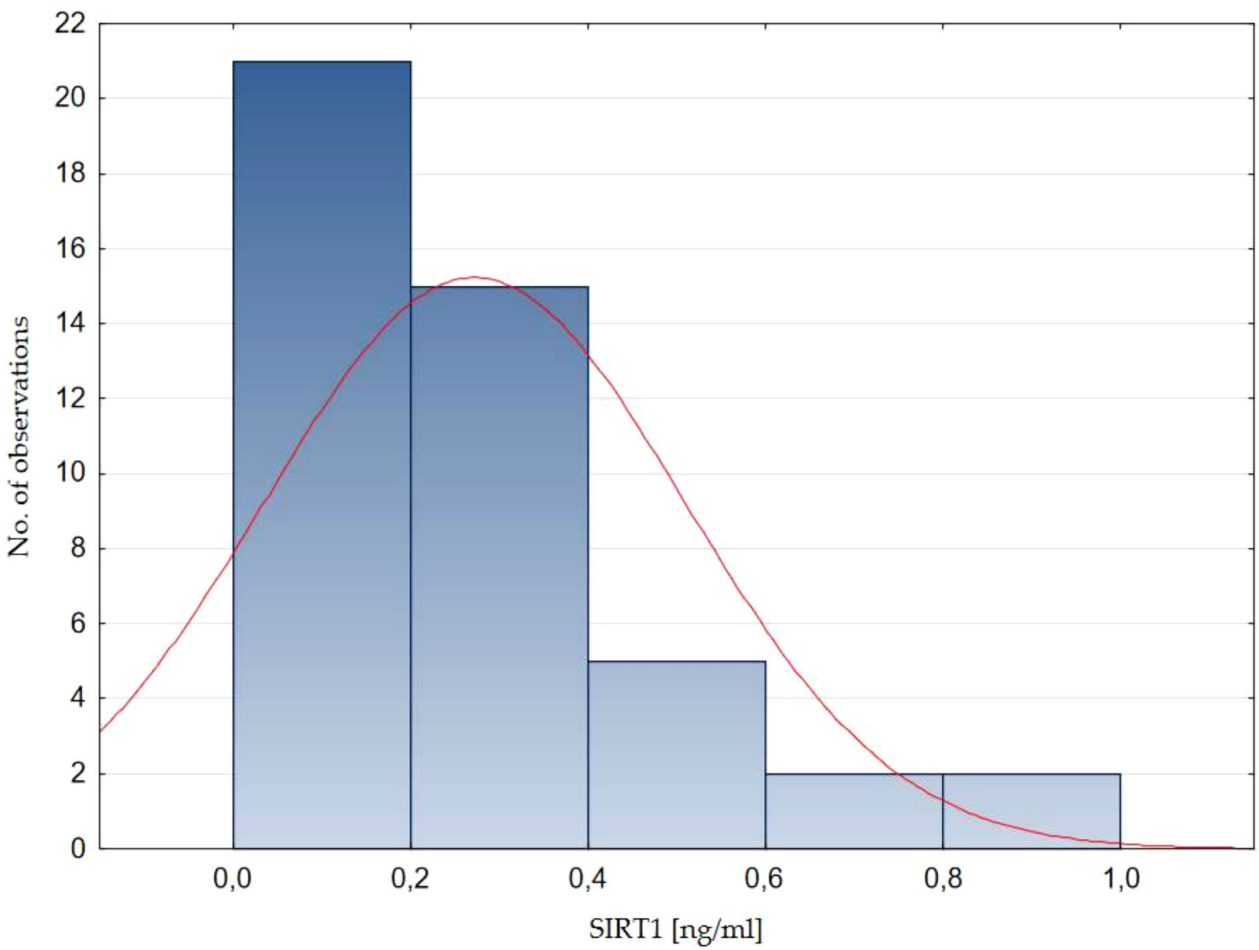
Distribution of sirtuin 1 concentration in a group of healthy children. SIRT1, sirtuin 1.

Serum SIRT1 concentration in healthy children ranged from 0.04 to 0.96 ng/ml. The mean SIRT1 concentration in healthy children was 0.29 ± 0.21 ng/ml (mean ± SD), while the median value (Q1-Q3) was 0.26 (0.14-0.38) ng/ml. The normality of the distribution of SIRT1 concentration was assessed - no normal distribution was found (Shapiro - Wilk test, p=0.0002, **Figure 1**).

**Figure 2:**
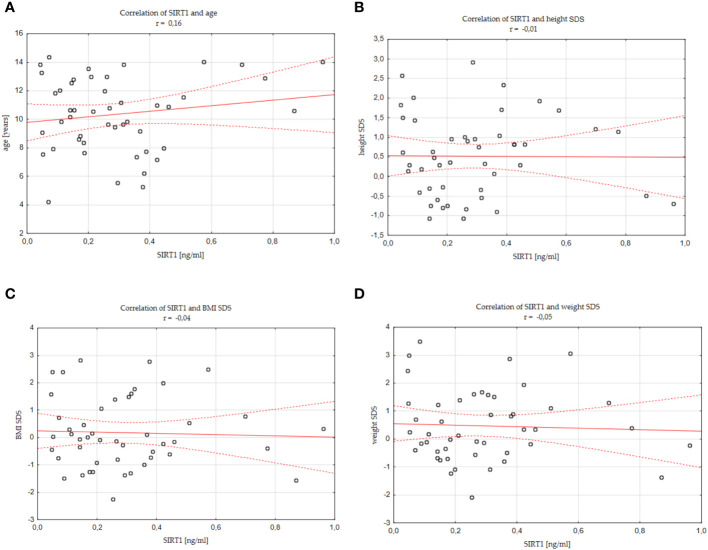
Correlations between SIRT1 concentration and individual analysed parameters **(A)** age, **(B)** height SDS, **(C)** BMI SDS, **(D)** weight SDS, standard deviation score; BMI, body mass index.

There was no significant correlation between SIRT1 concentration and age of children (r=0.16), SIRT1 concentration and weight of children (r=0.11), SIRT1 concentration and weight SDS values (r=-0.05), SIRT1 concentration and height of children (r=0.13), as well as SIRT1 concentration and height SDS values (r=-0.01), SIRT1 concentration and their BMI (r=0.05), SIRT1 concentration and BMI SDS values (r=-0.04). We also did not find correlations between SIRT1 and IGF-1 concentrations (r=0.11), SIRT1 concentration and IGF-1 SDS value (r=0.08), SIRT1 and IGFBP-3 concentrations (r=0.12), as well as between SIRT1 concentration and IGF-1/IGFBP-3 molar ratio (r=0.06). The mentioned results are presented in **Figure 2** and **Supplementary Figure 1**.

We also compared the SIRT1 concentration in individual subgroups of children, differentiated by gender (girls vs boys), age (younger than 10 years vs older or equal to 10 years old), stage of puberty (prepubertal vs pubertal), body weight and height (BMI SDS/hSDS greater or equal to 0 vs less than 0) and IGF1 concentration (IGF-1 SDS above or equal to 0 vs below 0). There were no statistical differences between SIRT 1 levels in the analysed subgroups (**Table 2**).

**Table 2:** SIRT1 concentration in healthy patients with respect to various parameters.

Variable	Variable	No	SIRT1 [ng/ml]	p
Sex	female	21	0.24 (0.15 – 0.38)	0.9
	male	26	0.26 (0.14 – 0.37)	
Age [years]	<10	21	0.28 (0.17 – 0.37)	0.84
	≥10	26	0.23 (0.14 – 0.46)	
Puberty [Tanner stage]	=1	24	0.29 (0.14 – 0.38)	0.52
	≥2	23	0.21 (0.14 – 0.37)	
BMI SDS	<0	24	0.26 (0.16 – 0.38)	0.55
	≥0	23	0.20 (0.10 – 0.38)	
height SDS	<0	15	0.20 (0.14 – 0.31)	0.9
	≥0	32	0.27 (0.1 – 0.41)	
IGF-1 SDS	<0	26	0.28 (0.14 – 0.37)	0.43
	≥0	21	0.26 (0.14 – 0.44)	

BMI, body mass index; SDS, standard deviation score; IGF-1, Insulin-like growth factor 1.

### 3.3 Dependence of SIRT1 concentration on the frequency of consumption of particular types of food

**Figure 3:**
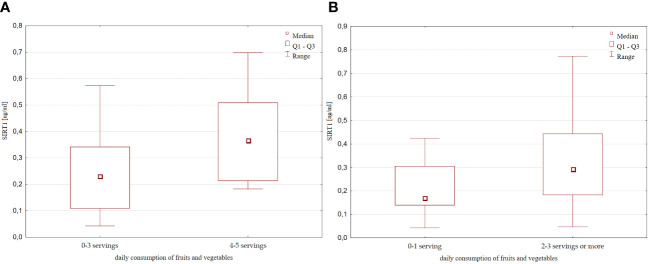
SIRT1 concentration with respect to the daily fruits and vegetable consumption **(A)** 0-3 servings vs 4-5 servings per day; **(B)** 0-1 serving vs 2-3 servings or more per day;.

Based on the declared daily fruits and vegatables intake estimated in the overview survey described in Materials and methods, we found that children who consumed at least 4-5 portions of fruits or vegetables per day had significantly higher levels of SIRT1 than children who consumed less (0-3 portions per day) [0.41 (0.23 – 0.6) ng/ml vs 0.2 (0.1 – 0.34) ng/ml, p=0.02, **Figure 3**]. A tendency towards higher SIRT1 concentration was also found in the group of children eating at least 2-3 or more servings of fruits and vegetables, compared to those eating 0 or a maximum of 1 serving per day [0.29 (0.18 – 0.44) ng/ml vs 0.17 (0.14 – 0.3) ng/ml, p=0.068].

**Figure 4:**
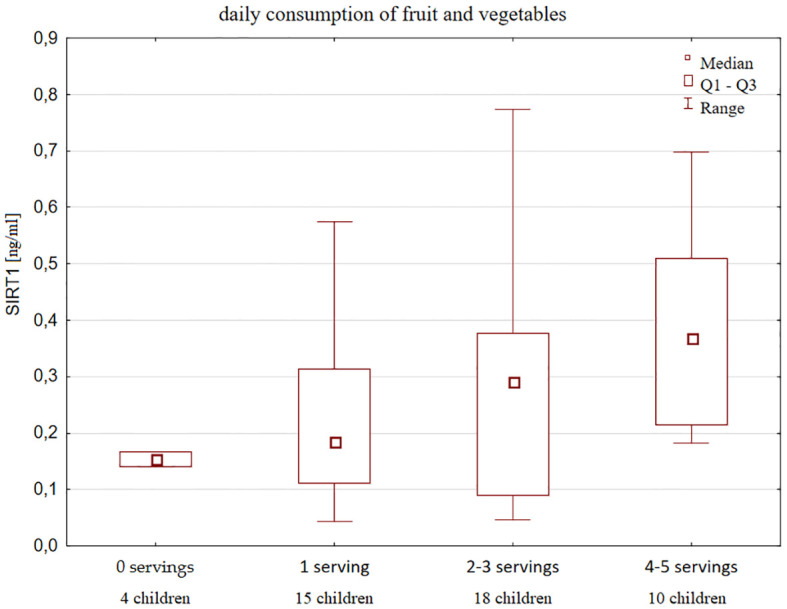
Serum SIRT1 concentration according to the declared frequency of fruits and vegetables intake in healthy children and a number of children in each group.

Moreover, SIRT1 concentration increased with declared frequency of fruits and vegetables consumption (**Figure 4**).

**Figure 5:**
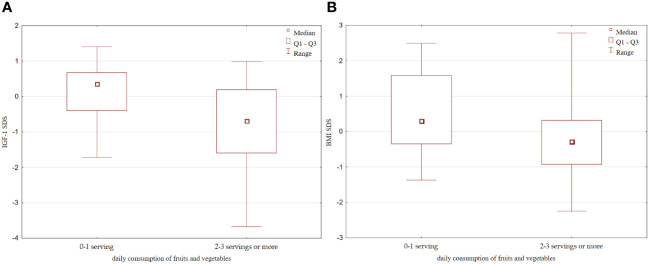
Differences in: **(A)** IGF-1 SDS; **(B)** BMI SDS with respect to the daily fruits and vegetables consumption. IGFF-1, Insulin-like growth factor 1; IGFBP-3, insulin-like growth factor binding protein-3.

Although no correlation was observed between SIRT1 and IGF-1 levels in the whole analysed group, after dividing children into groups according to the amount of vegetables and fruits consumed, we found that children eating more fruits and vegetables (at least 2-3 servings per day), despite higher levels of SIRT1, had also significantly lower IGF-1 concentration ([149.6 (87.4 – 349.8) ng/ml vs 379 (156.2 – 502.3) ng/ml, p=0.01, **Figure 5**], and IGF-1 SDS values [-0.69 (-1.59 – 0.2) vs 0.37 (-0.4 – 0.68), p=0.006, **Figure 5**], as well as decreased IGF-1/IGFBP-3 molar ratio [0.22 (0.18 – 0.38) vs 0.4 (0.22 – 0.5), p=0.02]. Furthermore, those children were found to have lower BMI [16.64 (15 – 18.67) vs 18.24 (16.22 – 21.2), p=0.056, **Figure 5**] and BMI SDS [-0.39 (-0.92 – 0.31) vs 0.3 (-0.34 – 1.6), p=0.088, **Figure 5**]. Nevertheless, they did not differ with respect to height, gender and age.

**Figure 6:**
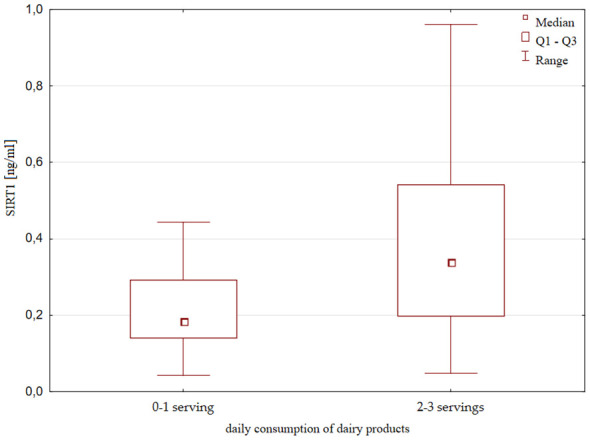
Serum SIRT1 concentration in terms of dairy products consumption.

SIRT1 concentration was also slightly but significantly higher in the group of children consuming unsweetened dairy products more frequently; that is, with consumption at least 2-3 times a day compared to consumption of 0-1 once a day [0.34 (0.2 – 0.54) ng/ml vs 0.18 (0.14 – 0.29) ng/ml, p=0.018, **Figure 6**]. There were no significant differences in height, weight and IGF-1 concentration with respect to the frequency of the consumption of dairy products.

No differences in SIRT1 levels were detected with respect to the consumption frequency of bread, rice, pasta, fish, meat, sweets and sugary drinks, nor in relation to a specific diet or a number of daily meals. Data on those individual food groups that were compared in the survey are included in the **Supplementary Materials**.

## 4 Discussion

SIRT1 is a protein that is primarily localized intracellularly (in a nucleus of liver, muscle, and white adipose tissue and in a cytoplasm of pancreatic and endothelial cells) (3, 5). It has been detected in the human adult serum (33) and its concentration appears to be altered in various disease states. SIRT1 serum reduction was shown in Alzheimer’s disease and mild cognitive impairment (33), obesity (34) and lung diseases (35), suggesting that serum SIRT1 may be a potential biomarker for various aging-associated diseases. On the other hand, increased serum SIRT1 levels were noticed in acute ischemic stroke (36), asthma (37) systemic lupus erythematosus (38) and frailty (39). To quantify SIRT1 levels in serum samples enzyme-linked immunosorbent assays (ELISA) technique was used most frequently.

To our knowledge, this study represents the first attempt to assess the serum concentration of SIRT1 in children and to explore the potential influencing factors. When compared to studies conducted in the adult population, the levels of SIRT1 observed in children within our study exhibited slightly lower concentration (35, 37, 40–43). There was one earlier study (44) focusing on SIRT1 levels in children in the context of growth. In this study SIRT1 levels were notably higher than the values reported in our observation. However, the authors of the cited report presented a small sample size with only male children and did not refer to other potential factors that may influence SIRT1 concentration in a population of healthy children (44). Nevertheless, the observed differences in serum concentration could be attributed to variations in the assay sources or the laboratory techniques (35, 37, 40–43).

It was proven that, SIRT1 play an important role in detection of cellular energy levels and regulation of energy metabolism and increase in response to fasting, caloric restriction and malnutrition (3). Several reports have shown that serum SIRT1 levels correlated negatively with BMI and were elevated in patients with anorexia nervosa (1, 45). It is well known that caloric restriction contributes to a state of growth hormone resistance and is associated with a decrease in serum IGF-1 levels, which is a main mediator of growth hormone (GH) action in peripheral tissues. SIRT1 is known to influence the process of intracellular GH signal transduction for IGF-1 synthesis (46). In situations of fasting or nutrient deficiencies, SIRT1 has been found to decrease the release of IGF-1 from the liver through the STAT5 pathway and enhance resistance to GH by promoting the release of GH from the pituitary gland (13, 47, 48). Furthermore, the presence of SIRT1 in the hypothalamus, particularly in neurons expressing the growth hormone receptor (GHR), and in chondrocytes, suggests potential relevance to the context of growth regulation (12, 14, 15, 49). It has been also suggested that SIRT1 regulate kisspeptin expression in the hypothalamus, affecting the timing of puberty onset (20). Therefore, it seemed to us that SIRT1 levels might vary depending on child height, or pubertal stage. However, we did not observe any significant differences in serum SIRT1 levels in relation to, age, height and IGF-1 levels as well as pubertal development. Although it has been found that SIRT1 levels decrease with age (50), in our paediatric cohort with a limited age range these differences may not be apparent.

As our results did not reflect associations between SIRT1 serum levels and height, weight or puberty, the intracellular function of SIRT1 may be defined by factors other than blood concentration. However, taking into account data from the literature, further investigation regarding SIRT1 involvement in growth disorders as well as weight and pubertal disturbances is warranted.

As SIRT1 is engaged in responding to metabolic imbalances, the amount of SIRT1 depends on the availability and type of nutrients (51). We assessed that SIRT1 serum levels were higher in children that consume more fruits and vegetables (at least 2-3 portions per day). Interestingly, those children were thinner and had lower IGF-1 serum levels, whereas their height was not affected the reduced IGF-1 serum levels may be related to sirtuins (46), but too many factors affect IGF-1 values to draw conclusions on this topic. Our outcomes are consistent with studies showing that sirtuin 1 may be activated by certain polyphenols, a class of naturally-occurring phytochemicals, which are compounds of some dietary products - fruits and vegetables in particular. The best known SIRT1 activator, resveratrol can be found in grapes, blueberries and grape products such as red wine (21, 23, 51–53). Piceatannol is a metabolite of resveratrol detected in grapes, passion fruit and white tea (23, 24). Quercetin, flavonoid polyphenol is present in fruits (peaches), vegetables (onions, garlic) and nuts (22, 54). Other dietary polyphenols that activates SIRT1 is fisetin, which can be found in apples, kiwi, dactyls, strawberries and blueberries among others (22, 53, 55). Some data suggest that besides polyphenols, dairy components may also serve as SIRT1 activators. Regarding our results, children with high consumption of dairy products had significantly increased SIRT1 serum concentration. Correspondingly*, in vitro* study in muscle and adipose cells indicate that systemic effects of high dairy feeding resulted in increased SIRT1 gene expression and activity in those cells (56). In addition, leucine, which is present in dairy food was proven to increase SIRT1 expression (57). Despite studies indicate that both natural and synthetic sirtuin activating compounds increase SIRT1 activity *in vivo*, the precise mechanism by which they activate SIRT1 remains unclear (58, 59). According to data from the literature resveratrol and others sirtuin 1 activators have demonstrated promising outcomes in a wide range of age-related diseases including obesity, diabetes, inflammation, cardiovascular disease, among others (60–64). Huang et al. summarized the current experience regarding resveratrol treatment in people with obesity, finding a significant improvement in metabolic complications and body weight reduction (65). A favourable effect of resveratrol has also been shown in certain diseases in children, such as ADHD or muscular dystrophies (66, 67).

Sirtfood is a novel food concept, according to which compounds from diet can affect sirtuins (53). Sirtfoods are said to induce a calorie restriction state and reduce nutrient consumption without causing malnutrition (68). The vast majority of data show that foods containing sirtuins (Sirtfoods) may produce pleiotropic, beneficial effects on health, alleviating metabolic disorders (53). Formulating a dietary regimen that integrates sirtuin-activating components derived from both the Asian and Mediterranean diets presents a potentially efficacious strategy in the prevention of chronic diseases. This approach holds promise for fostering health and supporting healthy aging (69). However, low bioavailability and rapid metabolism of polyphenols are issues that need to be scientifically addressed (70, 71). Further research is needed to evaluate dietary impact on sirtuin 1 and Sirtfoods-associated clinical implications.

Despite our study is limited by a small sample size, it is important to highlight that it represents the first investigation focusing on assessing SIRT1 serum levels in children which may offer valuable insights into the role of SIRT1 in pediatric physiology and its potential relevance to various aspects of child health. The preliminary findings presented here may serve as a basis for further research in this area and contribute to our understanding of SIRT1 implications in childhood development and disease, with an emphasis on growth and nutrition. Future studies with larger sample sizes and a wider age range are warranted to validate and expand upon the findings of this pioneering investigation.

## 5 Conclusions/highlights

1. Serum sirtuin 1 concentrations in healthy children did not differ with respect to sex, age, pubertal development, axiological parameters and IGF-1 levels.
2. Higher frequency of fruits, vegetables and dairy products consumption appeared to increase serum sirtuin 1 levels.

## Data availability statement

The raw data supporting the conclusions of this article will be made available by the authors, without undue reservation.

## Ethics statement

The studies involving humans were approved by Bioethics Committee at the Polish Mother’s Memorial Hospital – Research Institute in Lodz (Opinion No. 47/2020). The studies were conducted in accordance with the local legislation and institutional requirements. Written informed consent for participation in this study was provided by the participants’ legal guardians/next of kin.

## Author contributions

AF: Conceptualization, Data curation, Funding acquisition, Investigation, Methodology, Project administration, Resources, Software, Validation, Visualization, Writing – original draft, Writing – review & editing. AL: Supervision, Writing – review & editing. RS: Conceptualization, Formal analysis, Funding acquisition, Investigation, Methodology, Project administration, Supervision, Validation, Visualization, Writing – review & editing.

## References

[1] Haigis MC Sinclair DA. Mammalian sirtuins: b iological insights and disease relevance. Annu Rev Pathol: Mech Dis. (2010) 5:253–95. doi: 10.1146/annurev.pathol.4.110807.092250 PMC2866163 20078221

[2] Bonkowski MS Sinclair DA. Slowing ageing by design: the rise of NAD+ and sirtuin-activating compounds. Nat Rev Mol Cell Biol. (2016) 17:679–90. doi: 10.1038/nrm.2016.93 PMC5107309 27552971

[3] Nogueiras R Habegger KM Chaudhary N Finan B Banks AS Dietrich MO. SIRTUIN 1 AND SIRTUIN 3: PHYSIOLOGICAL MODULATORS OF METABOLISM. Physiol Rev. (2012) 92:1479–514. doi: 10.1152/physrev.00022.2011 PMC3746174 22811431

[4] Hwang Jw Yao H Caito S Sundar IK Rahman I. Redox regulation of SIRT1 in inflammation and cellular senescence. Free Radic Biol Med. (2013) 61:95–110. doi: 10.1016/j.freeradbiomed.2013.03.015 23542362 PMC3762912

[5] Xie L Huang R Liu S Wu W Su A Li R. A positive feedback loop of SIRT1 and miR17HG promotes the repair of DNA double-stranded breaks. Cell Cycle. (2019) 18:2110–23. doi: 10.1080/15384101.2019.1641388 PMC6986557 31290724

[6] Alves-Fernandes DK Jasiulionis MG. The role of SIRT1 on DNA damage response and epigenetic alterations in cancer. Int J Mol Sci. (2019) 20:3153. doi: 10.3390/ijms20133153 31261609 PMC6651129

[7] Kauppinen A Suuronen T Ojala J Kaarniranta K Salminen A. Antagonistic crosstalk between NF-κB and SIRT1 in the regulation of inflammation and metabolic disorders. Cell Signal. (2013) 25:1939–48. doi: 10.1016/j.cellsig.2013.06.007 23770291

[8] Pang J Xiong H Ou Y Yang H Xu Y Chen S. SIRT1 protects cochlear hair cell and delays age-related hearing loss via autophagy. Neurobiol Aging. (2019) 80:127–37. doi: 10.1016/j.neurobiolaging.2019.04.003 31170533

[9] Zhou S Tang X Chen HZ. Sirtuins and insulin resistance. Front Endocrinol (Lausanne). (2018) 9:748. doi: 10.3389/fendo.2018.00748 30574122 PMC6291425

[10] Yang Y Liu Y Wang Y Chao Y Zhang J Jia Y. Regulation of SIRT1 and its roles in inflammation. Front Immunol. (2022) 13:831168. doi: 10.3389/fimmu.2022.831168 35359990 PMC8962665

[11] Kitada M Ogura Y Monno I Koya D. Sirtuins and type 2 diabetes: role in inflammation, oxidative stress, and mitochondrial function. Front Endocrinol (Lausanne). (2019) 10:187. doi: 10.3389/fendo.2019.00187 30972029 PMC6445872

[12] Kang X Yang W Wang R Xie T Li H Feng D. Sirtuin-1 (SIRT1) stimulates growth-plate chondrogenesis by attenuating the PERK-eIF-2α-CHOP pathway in the unfolded protein response. J Biol Chem. (2018) 293:8614–25. doi: 10.1074/jbc.M117.809822 PMC5986218 29653943

[13] Yamamoto M Iguchi G Fukuoka H Suda K Bando H Takahashi M. SIRT1 regulates adaptive response of the growth hormone–insulin-like growth factor-I axis under fasting conditions in liver. Proc Natl Acad Sci USA. (2013) 110:14948–53. doi: 10.1073/pnas.1220606110 PMC3773795 23980167

[14] de Lima JBM Ubah C Debarba LK Ayyar I Didyuk O Sadagurski M. Hypothalamic GHR-SIRT1 axis in fasting. Cells. (2021) 10:891. doi: 10.3390/cells10040891 33919674 PMC8069818

[15] Satoh A Brace CS Ben-Josef G West T Wozniak DF Holtzman DM. SIRT1 promotes the central adaptive response to diet restriction through activation of the dorsomedial and lateral nuclei of the hypothalamus. J Neurosci. (2010) 30:10220–32. doi: 10.1523/JNEUROSCI.1385-10.2010 PMC2922851 20668205

[16] Ramadori G Fujikawa T Fukuda M Anderson J Morgan DA Mostoslavsky R. SIRT1 deacetylase in POMC neurons is required for homeostatic defenses against diet-induced obesity. Cell Metab. (2010) 12:78–87. doi: 10.1016/j.cmet.2010.05.010 20620997 PMC2904327

[17] Opstad TB Sundfør T Tonstad S Seljeflot I. Effect of intermittent and continuous caloric restriction on Sirtuin1 concentration depends on sex and body mass index. Nutr Metab Cardiovasc Dis. (2021) 31:1871–8. doi: 10.1016/j.numecd.2021.03.005 33975734

[18] Gillum MP Erion DM Shulman GI. Sirtuin-1 regulation of mammalian metabolism. Trends Mol Med. (2011) 17:8–13. doi: 10.1016/j.molmed.2010.09.005 20971038 PMC3123438

[19] Bordone L Guarente L. Calorie restriction, SIRT1 and metabolism: understanding longevity. Nat Rev Mol Cell Biol. (2005) 6:298–305. doi: 10.1038/nrm1616 15768047

[20] Vazquez MJ Toro CA Castellano JM Ruiz-Pino F Roa J Beiroa D. SIRT1 mediates obesity- and nutrient-dependent perturbation of pubertal timing by epigenetically controlling Kiss1 expression. Nat Commun. (2018) 9:4194. doi: 10.1038/s41467-018-06459-9 30305620 PMC6179991

[21] Gertz M Nguyen GTT Fischer F Suenkel B Schlicker C Fränzel B. A molecular mechanism for direct sirtuin activation by resveratrol. PloS One. (2012) 7:e49761. doi: 10.1371/journal.pone.0049761 23185430 PMC3504108

[22] Iside C Scafuro M Nebbioso A Altucci L. SIRT1 activation by natural phytochemicals: an overview. Front Pharmacol. (2020) 11:1225. doi: 10.3389/fphar.2020.01225 32848804 PMC7426493

[23] Dai H Sinclair DA Ellis JL Steegborn C. Sirtuin activators and inhibitors: Promises, achievements, and challenges. Pharmacol Ther. (2018) 188:140–54. doi: 10.1016/j.pharmthera.2018.03.004 PMC6342514 29577959

[24] Piotrowska H Kucinska M Murias M. Biological activity of piceatannol: Leaving the shadow of resveratrol. Mutat Res/Reviews Mutat Res. (2012) 750:60–82. doi: 10.1016/j.mrrev.2011.11.001 22108298

[25] Kułaga Z Grajda A Gurzkowska B Góźdź M Wojtyło M Swiąder A. Polish 2012 growth references for preschool children. Eur J Pediatr. (2013) 172:753–61. doi: 10.1007/s00431-013-1954-2 PMC3663205 23371392

[26] Kułaga Z Litwin M Tkaczyk M Palczewska I Zajączkowska M Zwolińska D. Polish 2010 growth references for school-aged children and adolescents. Eur J Pediatr. (2011) 170:599–609. doi: 10.1007/s00431-010-1329-x 20972688 PMC3078309

[27] Tanner JM Whitehouse RH. Clinical longitudinal standards for height, weight, height velocity, weight velocity, and stages of puberty. Arch Dis Child. (1976) 51:170–9. doi: 10.1136/adc.51.3.170 PMC1545912 952550

[28] Elmlinger MW Kühnel W Weber MM Ranke MB. Reference ranges for two automated chemiluminescent assays for serum insulin-like growth factor I (IGF-I) and IGF-binding protein 3 (IGFBP-3). Clin Chem Lab Med. (2004) 42:654–64. doi: 10.1515/CCLM.2004.112 15259383

[29] Haj-Ahmad LM Mahmoud MM Sweis NWG Bsisu I Alghrabli AM Ibrahim AM. Serum IGF-1 to IGFBP-3 molar ratio: A promising diagnostic tool for growth hormone deficiency in children. J Clin Endocrinol Metab. (2023) 108(4):986–94. doi: 10.1210/clinem/dgac609 36251796

[30] Poulain T Spielau U Vogel M Körner A Kiess W. CoCu: A new short questionnaire to evaluate diet composition and culture of eating in children and adolescents. Clin Nutr. (2019) 38:2858–65. doi: 10.1016/j.clnu.2018.12.020 30616881

[31] Jarosz M. Nowa Piramida Zdrowego Żywienia i Stylu Życia Dzieci i Młodzieży. Żyw Człow Meta. (2019) 46(1):13–4. Available at: https://ncez.pzh.gov.pl/dzieci-i-mlodziez/piramida-zdrowego-zywienia-i-stylu-zycia-dzieci-i-mlodziezy-2/.

[32] Kowalkowska J Wadolowska L Hamulka J Wojtas N Czlapka-Matyasik M Kozirok W. Reproducibility of a short-form, multicomponent dietary questionnaire to assess food frequency consumption, nutrition knowledge, and lifestyle (SF-FFQ4PolishChildren) in Polish children and adolescents. Nutrients. (2019) 11:2929. doi: 10.3390/nu11122929 31816859 PMC6950380

[33] Kumar R Chaterjee P Sharma PK Singh AK Gupta A Gill K. Sirtuin1: A promising serum protein marker for early detection of Alzheimer’s disease. PloS One. (2013) 8:e61560. doi: 10.1371/journal.pone.0061560 23613875 PMC3628714

[34] Mariani S Fiore D Persichetti A Basciani S Lubrano C Poggiogalle E. Circulating SIRT1 increases after intragastric balloon fat loss in obese patients. Obes Surg. (2016) 26:1215–20. doi: 10.1007/s11695-015-1859-4 26337692

[35] Yanagisawa S Papaioannou AI Papaporfyriou A Baker JR Vuppusetty C Loukides S. Decreased serum sirtuin-1 in COPD. Chest. (2017) 152:343–52. doi: 10.1016/j.chest.2017.05.004 PMC5540026 28506610

[36] Liu Y Jia S Liang X Dong M Xu X Lu C. Prognostic value of Sirtuin1 in acute ischemic stroke and its correlation with functional outcomes. Med (Baltimore). (2018) 97:e12959. doi: 10.1097/MD.0000000000012959 PMC6310560 30544370

[37] Wang Y Li D Ma G Li W Wu J Lai T. Increases in peripheral SIRT1: a new biological characteristic of asthma. Respirol. (2015) 20:1066–72. doi: 10.1111/resp.12558 26040995

[38] Yang C Li R Xu WD Huang AF. Increased levels of sirtuin-1 in systemic lupus erythematosus. Int J Rheumatic Dis. (2022) 25:869–76. doi: 10.1111/1756-185X.14360 35644944

[39] MaLNiuHShaGZhangYLiuPLiY. Serum SIRT1 is associated with frailty and adipokines in older adults. J Nutrition Health Aging. (2019) 23:246–50. doi: 10.1007/s12603-018-1149-7 30820512

[40] Gonçalinho GHF Nascimento JR de O Mioto BM Amato RV Moretti MA Strunz CMC. Effects of coffee on sirtuin-1, homocysteine, and cholesterol of healthy adults: does the coffee powder matter? J Clin Med. (2022) 11:2985. doi: 10.3390/jcm11112985 35683374 PMC9181040

[41] Charążka B Siejka A. Correlations between serum sirtuin levels and cardiovascular risk factors in women with polycystic ovary syndrome. Adv Med Sci. (2022) 67:123–8. doi: 10.1016/j.advms.2022.01.004 35134601

[42] Ozkan Kurtgoz P Karakose S Cetinkaya CD Erkus E Guney I. Evaluation of sirtuin 1 (SIRT1) levels in autosomal dominant polycystic kidney disease. Int Urol Nephrol. (2022) 54:131–5. doi: 10.1007/s11255-021-02862-2 33864594

[43] Mansur AP Roggerio A Goes MFS Avakian SD Leal DP Maranhão RC. Serum concentrations and gene expression of sirtuin 1 in healthy and slightly overweight subjects after caloric restriction or resveratrol supplementation: A randomized trial. Int J Cardiol. (2017) 227:788–94. doi: 10.1016/j.ijcard.2016.10.058 28029409

[44] Kaplan DS Canak A Isık E Orkmez M Kumru B. Relationship of fibroblast growth factor 21, sirtuin 1, visfatin, and regulators in children with short stature. Growth Factors. (2018) 36:172–7. doi: 10.1080/08977194.2018.1513504 30304969

[45] Amerio A Escelsior A Martino E Strangio A Aguglia A Marcatili M. The association between blood SIRT1 and ghrelin, leptin, and antibody anti-hypothalamus: A comparison in normal weight and anorexia nervosa. J Pers Med. (2023) 13:928. doi: 10.3390/jpm13060928 37373917 PMC10303472

[46] Fedorczak A Lewiński A Stawerska R. Involvement of sirtuin 1 in the growth hormone/insulin-like growth factor 1 signal transduction and its impact on growth processes in children. Int J Mol Sci. (2023) 24:15406. doi: 10.3390/ijms242015406 37895086 PMC10607608

[47] Fazeli PK Klibanski A. Determinants of GH resistance in malnutrition. J Endocrinol. (2014) 220:R57–65. doi: 10.1530/JOE-13-0477 PMC3994591 24363451

[48] Wójcik M Krawczyńska A Antushevich H Herman AP. Post-receptor inhibitors of the GHR-JAK2-STAT pathway in the growth hormone signal transduction. Int J Mol Sci. (2018) 19(7):1843. doi: 10.3390/ijms19071843 29932147 PMC6073700

[49] Yamamoto M Takahashi Y. The essential role of SIRT1 in hypothalamic-pituitary axis. Front Endocrinol (Lausanne). (2018) 9:605. doi: 10.3389/fendo.2018.00605 30405528 PMC6205959

[50] Zhong Y Chen AF Zhao J Gu YJ Fu GX. Serum levels of cathepsin D, sirtuin1, and endothelial nitric oxide synthase are correlatively reduced in elderly healthy people. Aging Clin Exp Res. (2016) 28:641–5. doi: 10.1007/s40520-015-0472-7 26462844

[51] Howitz KT Bitterman KJ Cohen HY Lamming DW Lavu S Wood JG. Small molecule activators of sirtuins extend Saccharomyces cerevisiae lifespan. Nature. (2003) 425:191–6. doi: 10.1038/nature01960 12939617

[52] Malaguarnera L. Influence of resveratrol on the immune response. Nutrients. (2019) 11:946. doi: 10.3390/nu11050946 31035454 PMC6566902

[53] Akan OD Qin D Guo T Lin Q Luo F. Sirtfoods: new concept foods, functions, and mechanisms. Foods. (2022) 11:2955. doi: 10.3390/foods11192955 36230032 PMC9563801

[54] Cui Z Zhao X Amevor FK Du X Wang Y Li D. Therapeutic application of quercetin in aging-related diseases: SIRT1 as a potential mechanism. Front Immunol. (2022) 13:943321. doi: 10.3389/fimmu.2022.943321 35935939 PMC9355713

[55] Khan N Syed DN Ahmad N Mukhtar H. Fisetin: a dietary antioxidant for health promotion. Antioxid Redox Signal. (2013) 19:151–62. doi: 10.1089/ars.2012.4901 PMC3689181 23121441

[56] Bruckbauer A Zemel MB. Effects of dairy consumption on SIRT1 and mitochondrial biogenesis in adipocytes and muscle cells. Nutr Metab (Lond). (2011) 8:91. doi: 10.1186/1743-7075-8-91 22185590 PMC3264668

[57] Li H Xu M Lee J He C Xie Z. Leucine supplementation increases SIRT1 expression and prevents mitochondrial dysfunction and metabolic disorders in high-fat diet-induced obese mice. Am J Physiol Endocrinol Metab. (2012) 303:E1234–44. doi: 10.1152/ajpendo.00198.2012 PMC3517633 22967499

[58] Hubbard BP Sinclair DA. Small molecule SIRT1 activators for the treatment of aging and age-related diseases. Trends Pharmacol Sci. (2014) 35:146–54. doi: 10.1016/j.tips.2013.12.004 PMC3970218 24439680

[59] Huang Y Lu J Zhan L Wang M Shi R Yuan X. Resveratrol-induced Sirt1 phosphorylation by LKB1 mediates mitochondrial metabolism. J Biol Chem. (2021) 297:100929. doi: 10.1016/j.jbc.2021.100929 34216621 PMC8326426

[60] Milne JC Lambert PD Schenk S Carney DP Smith JJ Gagne DJ. Small molecule activators of SIRT1 as therapeutics for the treatment of type 2 diabetes. Nature. (2007) 450:712–6. doi: 10.1038/nature06261 PMC2753457 18046409

[61] García-Martínez BI Ruiz-Ramos M Pedraza-Chaverri J Santiago-Osorio E Mendoza-Núñez VM. Effect of resveratrol on markers of oxidative stress and sirtuin 1 in elderly adults with type 2 diabetes. Int J Mol Sci. (2023) 24:7422. doi: 10.3390/ijms24087422 37108584 PMC10138491

[62] Li H Xia N Hasselwander S Daiber A. Resveratrol and vascular function. Int J Mol Sci. (2019) 20:2155. doi: 10.3390/ijms20092155 31052341 PMC6539341

[63] Najjar RS Feresin RG. Protective role of polyphenols in heart failure: molecular targets and cellular mechanisms underlying their therapeutic potential. Int J Mol Sci. (2021) 22:1668. doi: 10.3390/ijms22041668 33562294 PMC7914665

[64] Chen J Lou R Zhou F Li D Peng C Lin L. Sirtuins: Key players in obesity-associated adipose tissue remodeling. Front Immunol. (2022) 13:1068986. doi: 10.3389/fimmu.2022.1068986 36505468 PMC9730827

[65] Huang H Chen G Liao D Zhu Y Pu R Xue X. The effects of resveratrol intervention on risk markers of cardiovascular health in overweight and obese subjects: a pooled analysis of randomized controlled trials. Obes Rev. (2016) 17:1329–40. doi: 10.1111/obr.12458 27456934

[66] Kawamura K Fukumura S Nikaido K Tachi N Kozuka N Seino T. Resveratrol improves motor function in patients with muscular dystrophies: an open-label, single-arm, phase IIa study. Sci Rep. (2020) 10:20585. doi: 10.1038/s41598-020-77197-6 33239684 PMC7688653

[67] Rafeiy-Torghabeh M Ashraf-Ganjouei A Moradi K Bagheri S Mohammadi MR Akhondzadeh S. Resveratrol adjunct to methylphenidate improves symptoms of attention-deficit/hyperactivity disorder: a randomized, double-blinded, placebo-controlled clinical trial. Eur Child Adolesc Psychiatry. (2021) 30:799–807. doi: 10.1007/s00787-020-01562-z 32449130

[68] Lilja S Bäck H Duszka K Hippe B Suarez L Höfinger I. Fasting and fasting mimetic supplementation address sirtuin expression, miRNA and microbiota composition. Funct Foods Health Dis. (2020) 10:439. doi: 10.31989/ffhd.v10i10.752

[69] Pallauf K Giller K Huebbe P Rimbach G. Nutrition and healthy ageing: calorie restriction or polyphenol-rich ‘MediterrAsian’ diet? Oxid Med Cell Longev. (2013) 2013:707421. doi: 10.1155/2013/707421 24069505 PMC3771427

[70] Cueva C Silva M Pinillos I Bartolomé B Moreno-Arribas MV. Interplay between dietary polyphenols and oral and gut microbiota in the development of colorectal cancer. Nutrients. (2020) 12:625. doi: 10.3390/nu12030625 32120799 PMC7146370

[71] van Dorsten FA Grün CH van Velzen EJJ Jacobs DM Draijer R van Duynhoven JPM. The metabolic fate of red wine and grape juice polyphenols in humans assessed by metabolomics. Mol Nutr Food Res. (2010) 54:897–908. doi: 10.1002/mnfr.200900212 20013882

