# Supplementary material for: Sirtuin 1 serum concentration in healthy children - dependence on sex, age, stage of puberty, body weight and diet
Source: Front Endocrinol (Lausanne). 2024 Mar 11;15:1356612. doi: 10.3389/fendo.2024.1356612 (PMC10961438; doi:10.3389/fendo.2024.1356612)
Supplement: Supplementary file 1 [file DataSheet_1.docx]

Supplementary Material

# Supplementary Data: Questionnarie, English version (1) used with permission of the authors of the questionnaire

Full name of the child .......................................................................................................................

Date of birth ....................................................................................................................................

In the following questionnaire, you will be asked about your child's eating habits during the past 4 weeks. Please also include the food that your child eats out. Please mark the answers with an "X".


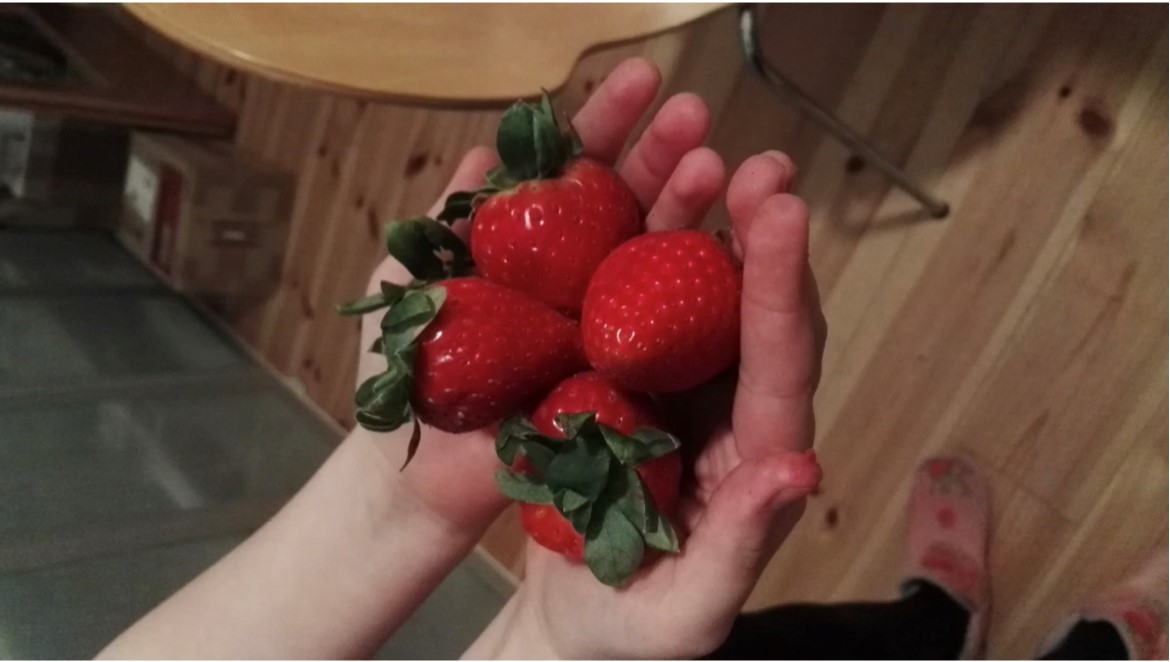


Figure 1: Please use the size of both of your child's hands as a reference.

| 1. How many portions ... does your child eat in ONE DAY? | No portion | max 1 serving | 2-3 | 4-5 | 6-7 | greater than 7 |
| --- | --- | --- | --- | --- | --- | --- |
| ... servings of vegetables/fruit (1 serving = as in Figure #1 or 1 apple, 1 pear, 1 kohlrabi, etc.). |  |  |  |  |  |  |
| ... servings of unsweetened dairy products (1 serving = 1 glass of milk, 150g cottage cheese, 150g natural yogurt, 1 slice of cheese) |  |  |  |  |  |  |
| ...servings of sweetened dairy products (1 serving = 150g pudding, 150g fruit yogurt, 1 cup cocoa) |  |  |  |  |  |  |
| ... glasses of sweetened beverages (e.g., lemonade, sweetened tea, flavored water) |  |  |  |  |  |  |
| ...servings of multigrain bread/rolls (1 serving = 1 slice of bread or 1 roll) |  |  |  |  |  |  |
| .... servings of white/dark bread/toast/white rolls (1 serving = 1 slice of bread or 1 roll) |  |  |  |  |  |  |


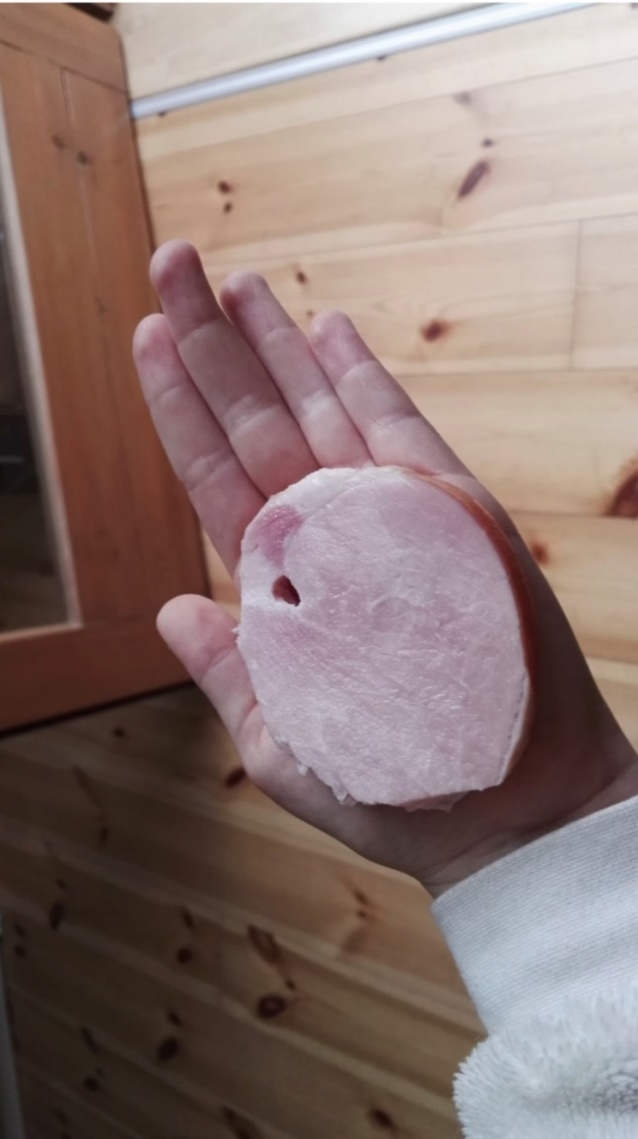


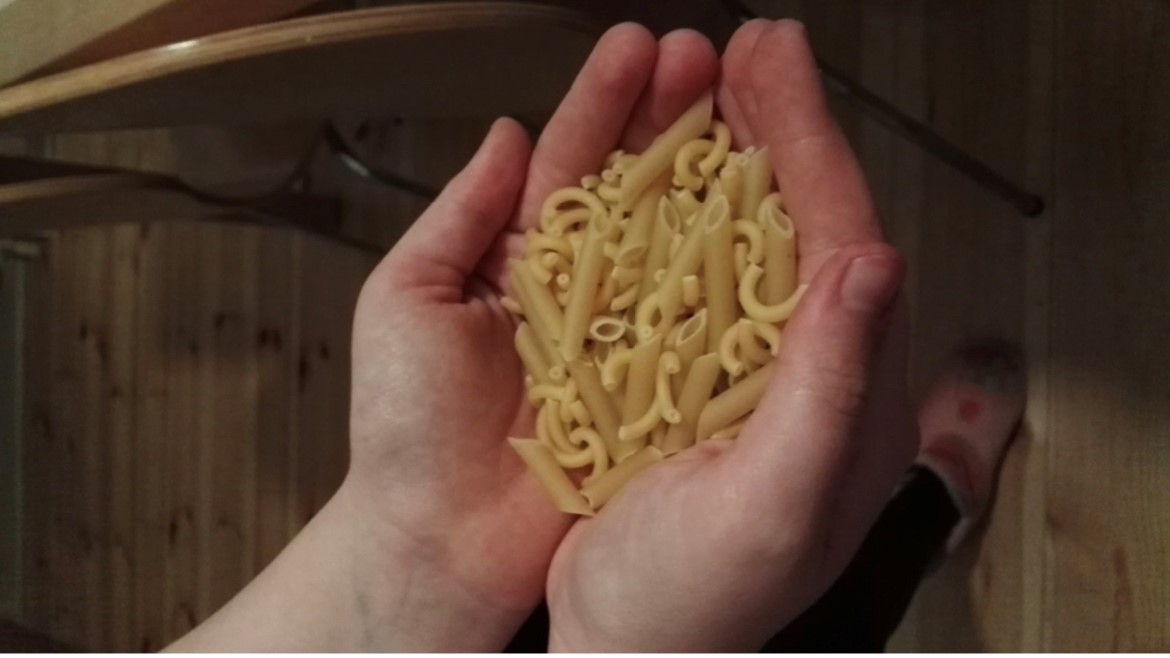


Figure 2* Figure 3*


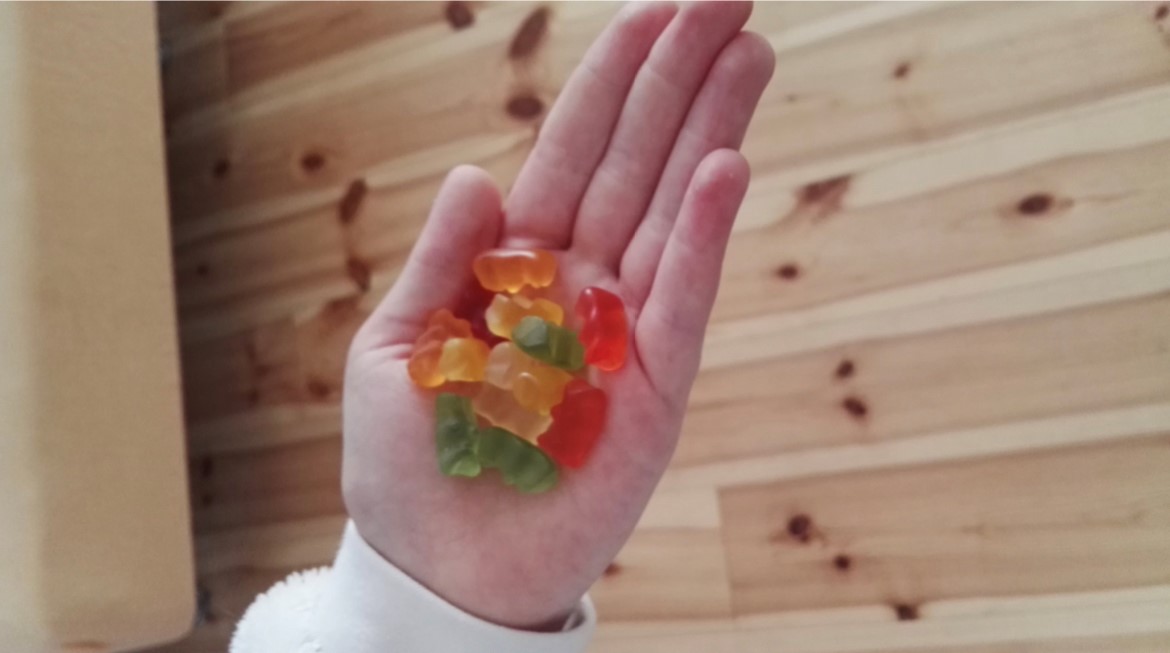


Figure 4*

*Please use your child's hand size as a reference.

| 1. How many portions ... does your child eat in ONE WEEK? | No portion | max 1 serving | 2-3 | 4-5 | 6-7 | greater than 7 |
| --- | --- | --- | --- | --- | --- | --- |
| ...portions of meat/steaks (1 portion = as in Figure #2 or 3 slices of sausage) |  |  |  |  |  |  |
| ...servings of fish (1 serving = as in Figure 2 or 1 canned fish, 3 fish sticks) |  |  |  |  |  |  |
| ...servings (as in Figure 3) of convenience foods such as frozen pizza, frozen pasta/lasagna, Chinese soup, microwaveable convenience foods |  |  |  |  |  |  |
| ...servings (as in Figure 3) of fried or baked potatoes, French fries |  |  |  |  |  |  |
| ...a serving (as in Figure 3) of rice or pasta |  |  |  |  |  |  |
| ...servings (as in Figure 3) of boiled potatoes |  |  |  |  |  |  |
| ...portions of cakes/cookies/yeast pastries (1 portion = 1 piece of cake, 3-4 cookies) |  |  |  |  |  |  |
| ...servings of sweet or salty snacks (1 serving = as in Figure 4 or 1 chocolate bar |  |  |  |  |  |  |

1. Does your child eat butter or margarine with bread/roll?
2. Yes, rather with reduced fat content
3. Yes, rather with normal fat content
4. Not
5. Does your child follow any specific diet?
6. Not
7. Yes, vegetarian
8. Yes, vegan
9. Yes, gluten-free or grain-free
10. Yes, lactose-free
11. Another - which one? ..............................................................
12. Which of the following meals does your child eat during the day?
13. Breakfast
14. Second breakfast
15. Lunch
16. Afternoon snack
17. Dinner
18. Does your child usually eat dinner with his/her parents?
19. Yes
20. Not
21. Is the TV or other media (tablet, cell phone) on at during dinner?
22. Yes
23. Not

1. Poulain T, Spielau U, Vogel M, Körner A, Kiess W. CoCu: A new short questionnaire to evaluate diet composition and culture of eating in children and adolescents. Clin Nutr. 2019 Dec;38(6):2858–65.
