# Supplementary figures and images for: Sirtuin 1 serum concentration in healthy children - dependence on sex, age, stage of puberty, body weight and diet
Source: Front Endocrinol (Lausanne). 2024 Mar 11;15:1356612. doi: 10.3389/fendo.2024.1356612 (PMC10961438; doi:10.3389/fendo.2024.1356612)

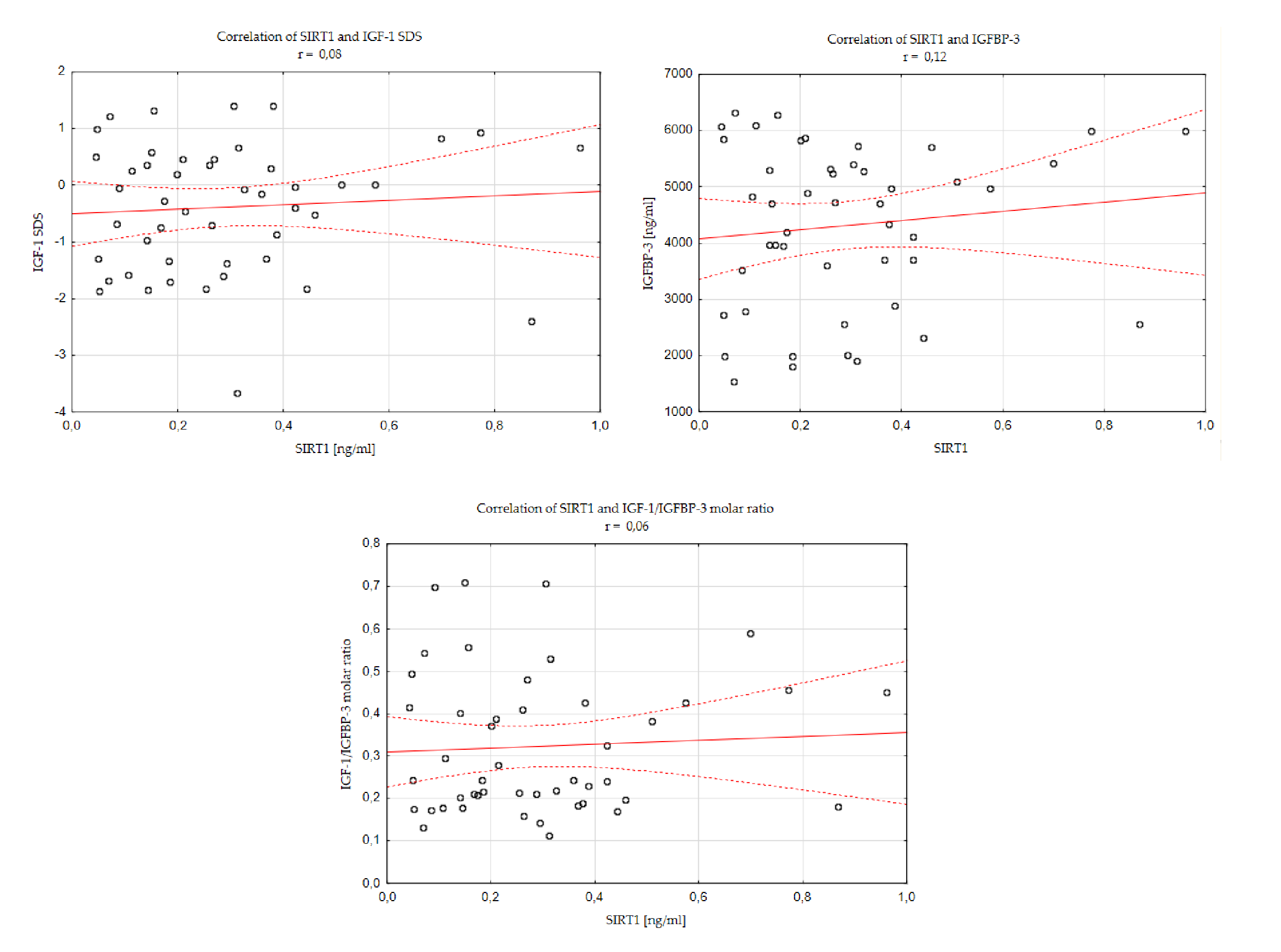

Supplement: Supplementary file 2 [file Image_1.tiff]
